# How individual social capital affects residents’ satisfaction with medical services: Based on the evidence from urban residents in China

**DOI:** 10.3389/fpsyg.2022.1077144

**Published:** 2022-12-09

**Authors:** Wenbin Wang, Yang Cao

**Affiliations:** Department of Sociology, Jilin University, Changchun, China

**Keywords:** satisfaction, medical services, social capital, Guanxi, medical resources, medical system

## Abstract

**Introduction:**

Residents’ satisfaction with medical services has commonly been treated as both a medical and psychosocial process. The influence of psychosocial factors on residents’ satisfaction with medical treatment is generally considered as important as that of medical factors. However, the effect of individual social capital on residents’ satisfaction after medical treatment–an important psychosocial variable that may influence health status and access to medical services–has not received sufficient attention.

**Methods:**

This study used the questionnaire survey data of urban residents in eight Chinese cities in 2014 to investigate how individual social capital affects residents’ satisfaction with medical services over the past year.

**Results:**

The results revealed a negative impact of individual social capital on residents’ overall satisfaction with medical services. In addition, the use of individual social capital significantly improved residents’ satisfaction with medical resources and significantly reduced residents’ satisfaction with the medical system. Moreover, the negative impact of individual social capital on residents’ overall satisfaction with medical services was greater for individuals with a lower likelihood of using this capital, which may lead to unequal allocation of medical resources and long-term life satisfaction.

**Discussion:**

The heterogeneous impact and mechanism of individual social capital on residents’ satisfaction with medical services was confirmed under the premise of self-selection bias.

## Introduction

Satisfaction towards medical services is a critical influencing factor for individuals to pursue a healthy and satisfying life. As a life event experienced by most people, the process and experience of seeking medical services are essential for maintaining the physical and mental health of the population. In addition to objective medical indicators such as medical expenses, waiting time, and medical services. Individual expectations and experiences of the medical treatment process may lead to different subjective evaluations, referred to as residents’ satisfaction with medical services (RSMS; Pascoe, 1983; Mosadeghrad, 2013). Just as health is considered an important area that constitutes overall life satisfaction according to the bottom-up theory of life satisfaction (Newman et al., 2014; Malvaso and Kang, 2022), RSMS is regarded as a subcomponent of health status and health-related quality of life which captures the impact of medical services on subjective well-being and life satisfaction (Lloyd, 2014). Different RSMS has a direct impact on residents’ current disease treatment and may affect residents’ overall confidence in future life (Cheng et al., 2003). Especially in developing countries like China, residents must engage in fierce competition and pay high costs to obtain limited medical resources, while lacking complete medical security, which has been shown to be significantly associated with lower resident happiness and life satisfaction (Kirchmann et al., 2013). This also means that RSMS may play a more important role in affecting the residents’ health status and future life expectancy of residents, especially in developing countries (Atkinson and Haran, 2005), and is a topic that requires more attention.

Under the condition of fierce competition for medical resources, social capital may become an important psychosocial factor for individuals to obtain medical resources and have a key impact on their RSMS. The influence of psychosocial factors including social capital on RSMS is generally considered as important as the influence of medical factors (Naidu, 2009; Victoor et al., 2012). In addition to accessibility and affordability of healthcare services (Zhang et al., 2021), psychological and social characteristics should also be taken into accounts, such as individual personality and expectations, interpersonal relations, and social capital (Bleich et al., 2009; Vinagre and Neves, 2010). Compared to medical factors, the influence strength and direction of psychosocial factors vary greatly in different societies (Batbaatar et al., 2017). Since high-quality medical resources are relatively scarce in most developing countries, individuals need to compete with others through various formal and informal means to obtain these resources. Therefore, an individual’s social network and social capital may become an important informal means to gain advantages in the competition for high-quality medical resources (Lu et al., 2022). Individual social capital may play a more important role in the access to medical resources and improvement of RSMS, especially in countries where formal medical systems and rules are not perfect, or residents are more dependent on social networks and social capital, such as in China (Bian, 2019). The outbreak of COVID-19 and the aging of the world population have created a strain on the medical supply and the growth of medical demand, respectively. In this context, the relevance and importance of individual social capital, medical resources and RSMS may also be increasing.

However, among the psychosocial factors that shape RSMS, the role of individual social capital embedded in individuals’ interpersonal relations is generally ignored (Raleigh et al., 2012; Batbaatar et al., 2017). A growing body of research has demonstrated the broad appeal of the social capital concept in explaining multiple phenomena in public health (Gilbert and Dean, 2013; Singh and Singh, 2020; West et al., 2022), such as the application of social capital in crisis management (Wang and Yunfeng, 2022)， or the intermediary role of social capital between mental health and satisfaction (Berthelsen et al., 2021). But social capital has been positioned at both the collective and individual levels in different research traditions. Social capital at the collective level refers to certain attributes, such as social group, social trust, and reciprocity, that can improve the life situation of group members (Fukuyama, 1995; Putnam, 1995). Social capital at the individual level is closely related to social networks and is defined as “resources embedded in a social structure that are accessed and/or mobilized in individuals’ purposive actions” (Lin, 2001). Individual and collective social capital have both been shown to have corresponding daily health consequences (Poortinga, 2006; Lagaert et al., 2021), including access to health resources, maintenance of mental health, and reduction of harmful behaviors, such as alcohol consumption, but only a few studies have linked social capital with residents’ satisfaction after medical treatment (Nieuwenhuis, 2020; Villalonga-Olives et al., 2020). Moreover, previous studies have defined social capital at the collective level, such as community participation, and security of residents and trust, cooperation, and justice of medical service providers (Kritsotakis et al., 2012; Nieuwenhuis, 2020). The influence on the RSMS of an individual’s social capital embedded in a social network remains poorly established.

China is considered a representative sample because the relationship between individual social capital and residents’ medical treatment behavior and RSMS becomes more important under the influence of Guanxi culture in Chinese society. Social capital is self-selected and maintained by individuals under certain social structures and is often associated with specific social situations and cultures working together (Pachucki and Breiger, 2010; Li and Guo, 2022). There is a wide culture of Guanxi in the Chinese social context. Individual social capital, formed through strong social ties, such as family and close friends, is generally regarded by Chinese residents as more trustworthy and reliable, providing full security and unconditional support (Bian, 1997; Bian, 2006). When Chinese residents are faced with difficulties or need to take action, individual social capital is often the preferred and sometimes even the necessary choice (Feng and Patulny, 2021; Zhang et al., 2021). Compared with residents in Western countries, Chinese residents have more dependence on and preference for individual social capital, especially social capital based on strong ties (Boissevain, 1974). Therefore, the use of Guanxi has become a cultural characteristic that influences the behavior of Chinese residents, also known as “Guanxi determinism” (Lin and Si, 2010). Moreover, individual social capital affects residents’ medical service seeking behaviors in China. Under the influence of Guanxi determinism, it is a common phenomenon in China to seek medical services through the operation of individual social capital (Zhang et al., 2021). Finding a familiar contact who may be able to help is seen by some residents as a requirement for better access to healthcare, although this may not be true. In addition to the advantages of resource acquisition usually shown by individual social capital, the spread of Guanxi culture may become a source of pressure for residents to seek medical treatment. This makes it more meaningful to understand individual social capital and RSMS in Chinese society, although complex challenges exist.

To date, only a few studies have explored this topic using qualitative methods. Although these studies have identified a close, often negative, association between individual social capital and RSMS (Zou et al., 2018; Zhang et al., 2021), there are still some theoretical and methodological shortcomings. Theoretically, the mechanism of individual social capital affecting RSMS remains unknown (Raleigh et al., 2012). Moreover, the effect of individual social capital on RSMS is undifferentiated across populations, which is hard to reconcile with the facts (Lin, 2001; Pachucki and Breiger, 2010). In terms of methodology, the self-selection bias in the use of social capital has not been fully considered. In addition, RSMS is usually measured as residents’ overall evaluation of the medical process. Residents may have different evaluations of medical resources and the medical system, which, respectively, reflect the professional and systematic characteristics of medical services. This topic needs further research.

We used the data from a large-scale questionnaire survey of urban residents in eight cities in China to investigate how individual social capital affects RSMS after medical treatment, and why this influence occurs. In particular, unlike previous studies which only measured the influence of individual social capital on overall RSMS, we measured the effect of individual social capital on residents’ satisfaction with medical resources and medical systems, and investigated the difference in the negative effect among people with varying likelihoods of using individual social capital. We then discussed the applicability of the social capital useless hypotheses, the social capital erosion hypotheses, and the social capital differentiation hypotheses in the field of healthcare services. Thus, we traced an informal psychosocial factor that may strongly influence RSMS under the premise of self-selection bias. Additionally, the heterogeneous impact and the mechanism of individual social capital on RSMS were also confirmed.

## Theoretical review and hypotheses development

### Measurement and determinants of residents’ satisfaction with medical services

The comprehensive and institutionalized modern medical service system has increased the complexity of RSMS. Although RSMS has become an important component of evaluating the healthcare system, there is a lack of agreement on exactly what to measure and how. The most common measurement indicators in early studies on RSMS involved doctor-patient relationships, medical quality, access to and convenience of medical resources, and medical costs (Ware et al., 1983). Although these indicators can completely reflect the residents’ subjective evaluation of specific medical services, they are essentially the evaluation of professional medical resources and ignore the possible institutionalization characteristics of RSMS (Tong et al., 2022). The modern medical system is no longer limited to the interaction between patients and doctors and is strictly constrained by various medical systems and regulatory authorities. Residents seeking medical services not only encounter internal professional risks, such as medical resources and costs, but also inevitably face the threat of external institutional risks, such as medical systems and safety (Giddens, 1991; Goold, 2001). Residents need to evaluate whether the medical service they receive is warm, convenient, effective, and affordable and whether abstract principles, such as medical safety and system, are correctly implemented, which constitutes an important source of RSMS. After receiving medical services, residents gain some new perceptions and evaluations of several issues, such as whether the existing medical insurance system ensures the medical needs of residents, whether medical resources are fairly and reasonably distributed in strict accordance with medical regulations, and whether medical resource service providers strictly follow the operation process to ensure medical safety. The evaluation of these institutional elements of medical services is not only an important component of RSMS but may also have a long-term impact on residents’ future medical treatment choices and life satisfaction.

Therefore, we need to pay attention to residents’ evaluation of various medical services during medical treatment using overall RSMS, as well as distinguish differences in satisfaction with the medical resources and system. The evaluation of medical resources, including expenses, is defined as residents’ satisfaction with medical resources, and the evaluation of abstract medical systems, such as medical safety and policy, is defined as residents’ satisfaction with the medical system. Residents’ satisfaction with medical resources and with the medical system can be complementary under certain conditions. Residents’ recognition of the medical system can improve the perceived value of the medical resources and promote satisfaction with these resources. Conversely, timely access to high-quality medical resources enhances residents’ confidence with the medical system (Zeithaml, 1988; Goold, 2001). However, substitution may be possible. When residents are generally dissatisfied with the medical system, high-quality medical services are more likely to improve residents’ satisfaction. Conversely, when residents are generally satisfied with the medical system, the evaluation criteria for satisfaction with medical resources are more stringent, and satisfaction with medical resources is difficult to improve (Zheng et al., 2017).

As with all satisfaction studies, the factors that influence RSMS are diverse and cannot be exhausted. They are often related to medical services and psychosocial factors. RSMS is generated in the continuous interaction between residents and medical service providers, and the performance of providers and the roles of residents are equally important (Donabedian, 1992). Healthcare provider-related determinants, such as service accessibility and outcome of care, have a consistent impact on RSMS in most studies. However, the strength of influence and direction of resident-related characteristics, such as individual attributes and social background, on RSMS vary greatly, and no consistent conclusion has been reached (Batbaatar et al., 2017). For instance, some studies have demonstrated a positive correlation between socioeconomic status and RSMS, while others have found no significant correlation or even a negative correlation (Vinagre and Neves, 2010; Footman et al., 2013). The same debate is taking place across the income spectrum. Higher income groups may be more satisfied with overall health services and access to medical resources, whereas lower income groups may be more satisfied with care (Ware Jr. et al., 1978; Xiao and Barber, 2008). Therefore, the formation of residents’ satisfaction is not only a medical but also a social process containing many complex psychosocial factors, which may show different characteristics under different social and cultural backgrounds (Wagner and Bear, 2009).

### Positive or negative: The dual effect of individual social capital on residents’ satisfaction with medical services

Due to scarce high-quality medical resources and extensive institutional uncertainty, individual social capital plays an important role in the acquisition of medical resources in Chinese society. However, this makes individual social capital a double-edged sword affecting RSMS. Previous studies have shown that in the field of intense resource competition and strong institutional uncertainty, individual social capital may play a more important role (Bian, 2019), and the process of medical services is in line with this characteristic. On the one hand, unlike in the United States and many developed countries in Europe, high-quality medical resources in China are relatively scarce and unevenly distributed. Experienced medical staff and advanced medical equipment are more concentrated in big cities and public hospitals. Due to limited medical resources, small city and private hospitals can only deal with basic diseases, such as colds and coughs. Moreover, as the supervision of public hospitals by the medical and health authorities is much stronger than that of private hospitals, the treatment process of public hospitals is stricter and safer. Therefore, although going to big cities and public hospitals is not convenient or cheap, it is likely to be a more satisfactory and reassuring option for residents, which further exacerbates the excessive competition for quality medical resources. On the other hand, the highly specialized threshold of medical care creates a general information asymmetry between residents and doctors, making residents highly dependent on doctors for medical information and medical countermeasures. In addition, China’s medical supervision and security systems are under continuous reform, and the institutional guarantee provided for residents as well as hospitals’ institutional supervision methods have changed in different years, which further increases the institutional uncertainty faced by residents. Therefore, the use of individual social capital has become common among residents in the process of medical treatment (Zou et al., 2018), and individual social capital has become a key social factor that may affect RSMS in China.

Just as individual social capital is not always positive for individual health, it may also have positive and negative effects on medical satisfaction. The positive view is that individual social capital can help residents obtain more scarce and high-quality medical resources. For instance, individuals undergoing medical treatment can recommend residents to hospitals with a better medical environment and more advanced medical equipment, refer residents to doctors with higher medical skills and more experience, and provide residents with lower and more preferential medical prices. Moreover, this may prompt medical staff to provide more suitable and warmer medical care to residents. These behaviors may help residents obtain more timely and satisfactory medical services, improving their overall satisfaction with medical services. However, the negative view notes that the use of individual social capital may not produce a deterministic advantage of medical resources, especially in public hospitals with a relatively complete medical system and strict medical supervision. Furthermore, the stakeholders may not be able to have a practical influence on the allocation of medical resources. Moreover, the use of individual social capital may damage the trust relationship between residents and doctors, increase residents’ unnecessary personal expenditures, and increase residents’ excessive expectations regarding medical services. Each of these consequences may lead to residents’ lower overall satisfaction with medical services. Several studies conducted in China provide some basis for the dual effect of Guanxi on medical satisfaction (Table 1).

**Table 1:** The role of individual social capital on the related indicators of RSMS in previous studies.

Dependent variable	Independent variable	Influence direction	Source	Year
Residents’ satisfaction	Seeking medical care through individual social capital	+ −	Cheng and Zou (2015)	2015
Medical satisfaction	Seeking medical services through individual social capital	−	Jianke and Jian (2017)	2017
Patient–physician trust	Seeking medical care through individual social capital	+ −	Zhou et al. (2018)	2018
Hospital trust	Using individual social capital	−	Li and Yang (2019)	2019
Doctor-patient relationship	Seeking medical services through individual social capital	−	Zhang et al. (2021)	2021

+ represents that the independent variable has a positive effect on the dependent variable, whereas − represents that the independent variable has a negative effect on the dependent variable.

Unlike the many positive functions of individual social capital in the business field and the labor market (Chung, 2011; Bian, 2018; Burt et al., 2018), the negative role of individual social capital on residents’ satisfaction is supported by more studies. Although some studies suggest that the use of individual social capital may bring certain benefits to individuals seeking medical services, this process is almost inevitably accompanied by corresponding negative consequences. Unfortunately, most studies are conducted using qualitative methods, which cannot provide a more representative analysis of the negative consequences of individual social capital. More importantly, it is difficult to control for the influence of self-selection bias, as the use of individual social capital does not occur randomly but is a highly self-selected process. People with different characteristics, such as income, gender, social network, or disease condition, may have different abilities and preferences in using individual social capital, which directly affects the possible consequences of using Individual social capital on medical satisfaction. This self-selection bias may lead to the misestimation of the utility of social capital, which needs special attention from social capital researchers (Mouw, 2003; Mouw, 2006). Therefore, the negative function of individual social capital on residents’ satisfaction requires further confirmation after controlling for self-selection bias. As such, the negative effect hypotheses were proposed:

*Hypothesis 1*: Individual social capital has a negative effect on overall satisfaction with medical service of urban residents, which is robust after considering self-selection bias.

*Hypothesis 1.1*: Urban residents who use individual social capital to find medical services have lower overall satisfaction with medical service.

*Hypothesis 1.2*: The negative effect of using individual social capital on residents’ overall satisfaction with medical service remains significant after controlling for self-selection bias.

### Useless, erosion, or differentiation: The mechanism of individual social capital on residents’ satisfaction with medical services

Under the premise of the emergence of the negative function of individual social capital, a noteworthy question is how this negative function occurs. Three self-consistent theoretical explanations have been formed around this problem: the useless hypothesis, the erosion hypothesis, and the differentiation hypothesis. However, in the current field of medical service, the mechanism of the negative function of individual social capital remains to be discussed.

The useless hypothesis doubts the rational basis of individual social capital, and believes that its use may be an irrational behavior guided by structural, cultural, and psychological characteristics, which does not necessarily produce positive benefits. Just as the returns of individual social capital in the labor market may be the result of the preference for homogeneous social communication (Mouw, 2003, 2006), the maintenance and investment of individual social capital may be affected by residents’ behavioral preferences and cultural traditions. For individuals, the cost of investing in individual social capital may not be valuable. It may not only be difficult to bring positive returns but may also become a burden. In China, individual social capital to obtain resources and services has been practiced for a long time and has become a behavioral preference recognized by many members of society (Yang, 1994; Gold et al., 2002; Ruan, 2017). The preferences and pressures of Guanxi determinism in Chinese society may attract or even force individuals to seek medical resources through individual social capital, although this may have no practical effect. As this view directly challenges the capital connotation of individual social capital, it has attracted considerable criticism (Lin and Ao, 2008; Chen and Volker, 2016).

According to the useless hypothesis, the negative impact of individual social capital on residents’ satisfaction may result from the pressure exerted by Guanxi culture in the process of medical service. Individual social capital may not bring real medical resource conveniences, such as better medical staff and better medical environment. However, for residents, the maintenance and use of individual social capital is indispensable. As other residents use individual social capital to seek medical services, if a resident does not use it, they may not only be excluded by other residents but may also be treated differently by doctors (Jianke and Jian, 2017; Zhang et al., 2021). Therefore, the use of individual social capital may be a forced behavior of residents, which not only cannot bring actual convenience for individuals but also increases the unnecessary economic and emotional burden on residents, creating painful medical experiences. Following this influence mechanism, the influence of individual social capital on residents’ satisfaction may be comprehensive and consistent, which impairs residents’ satisfaction not only with the medical system but also with medical resources. Therefore, the useless mechanism hypotheses were proposed:

*Hypothesis 2*: The negative effect of individual social capital on RSMS follows the useless mechanism.

*Hypothesis 2.1*: Residents who use individual social capital to seek medical services have lower satisfaction with medical resources.

*Hypothesis 2.2*: Residents who use individual social capital to seek medical services have lower satisfaction with the medical system.

Unlike the useless hypothesis, the erosion hypothesis suggests that individual social capital can bring resource advantages and positive experiences to individuals or small groups; however, this damages other groups and the system overall. According to this perspective, the operation of individual social capital may be affected by individual preferences and cultural pressures; however, this does not completely deny the possible resource advantages of social capital operation (Chen and Volker, 2016) despite often being a double-edged sword. Although close and closed social bonds and individual social capital provide sufficient emotional and resource support for some individuals, they may have negative consequences, such as exclusion of outsiders, excessive requirements for members, limitation of personal freedom, and reduction in norms (Portes, 1998). Studies on the process of healthcare seeking in China have demonstrated that seeking medical services through individual social capital improves resource convenience for residents, such as providing suitable and cheap medical plans and services. However, this operation, in which institutional procedures are replaced by personal bonds and general rules are replaced by specific regulations, may damage residents’ confidence in the overall medical system (Zou et al., 2018; Li and Yang, 2019).

According to the erosion hypothesis, the negative influence of individual social capital on RSMS comes from the erosion of the foundation of the existing medical system. Individual social capital can help individuals obtain better medical resources, pay lower medical costs, and achieve medical convenience. However, a possible violation of medical rules may damage residents’ confidence in overall system norms. Although individual social capital may be useful for residents, it may not be morally justifiable or suited to medical rules (Zou et al., 2018). Even if a resident obtains a better medical experience by using individual social capital, they may be more worried about the healthcare system in the future. In urgent situations with fierce competition for medical resources, people cannot be sure that they will be treated fairly by the medical system and not become a victim of the operation of individual social capital. As such, the influence of individual social capital on residents’ satisfaction with the medical system and resources may differ. Therefore, the erosion mechanism hypotheses were proposed:

*Hypothesis 3*: The negative effect of individual social capital on RSMS follows the erosion mechanism.

*Hypothesis 3.1*: Residents who use individual social capital to seek medical services have higher satisfaction with medical resources.

*Hypothesis 3.2*: Residents who use individual social capital to seek medical services have lower satisfaction with the medical system.

The differentiation hypothesis holds that the heterogeneous distribution of individual social capital in the population is also one of the causes of negative consequences. Under the constraints of social structure, the ability and cost of using individual social capital as well as the potential returns vary widely among different groups. For instance, people living in slums or poorly educated communities are often forced to search for work through homogeneous social networks in their communities due to a lack of other avenues for employment. A lack of non-repetitive information and resources in social networks greatly limits the help provided by social networks for slum residents and may even put workers in disadvantaged positions, causing social isolation and reproduction of poverty (Elliott, 1999; Wilson, 2012). Therefore, different use choices of individual social capital may cause significant differences in returns.

According to the differentiation hypothesis, the negative impact on RSMS is a result of the heterogeneous distribution of individual social capital. Based on the differences in socioeconomic characteristics and social network composition of residents, the availability and benefits of individual social capital differ. Residents with higher ability and likelihood of using individual social capital tend to have stronger social networks and action ability. The use of individual social capital is more likely to be their active choice after measuring costs and benefits, which is not only conducive to achieving the original goal of resource acquisition but can also maximize the avoidance of negative shocks brought by individual social capital.

Conversely, residents with a lower possibility of using individual social capital may have no social networks and action ability, and are the vulnerable group in the distribution of individual social capital. For them, the use of individual social capital means lower real value and higher mobilization cost, which is likely to be a passive choice made out of frustration. This blind choice without full consideration not only fails to bring actual medical resource convenience but may lead to a negative impression of the medical system and resources. Therefore, the negative impact of individual social capital on residents’ overall satisfaction with medical service may be weaker for residents with good individual social capital and amplified for residents lacking individual social capital. Therefore, the differentiation mechanism hypotheses were proposed:

*Hypothesis 4*: The negative effect of individual social capital on RSMS follows the differentiation mechanism.

*Hypothesis 4.1*: Compared to residents with a high possibility of using individual social capital, individual social capital has a greater negative effect on the overall satisfaction with medical service of residents with a low possibility of using it.

## Materials and methods

### Data sources

Data were obtained from the Social Network and Social Experience survey, which was conducted among urban residents in Changchun, Guangzhou, Jinan, Lanzhou, Shanghai, Tianjin, Xiamen, and Xi’an. The program was first initiated by the Institute for Empirical Social Science Research (IESSR) of Xi’an Jiaotong University in 2014 and jointly implemented by seven other universities across the country. The author’s research team was responsible for the survey in Changchun and obtained the right to use the national data. During the interview process, interviewers were asked to enter homes for face-to-face meetings with participants. The questionnaire was completed through Computer Assisted Personal Interviewing (CAPI), and a strict *post hoc* check was conducted to ensure the reliability and validity of the collected data. Participants were recruited through a strict multi-stage random sampling method by computer. First, we randomly selected the communities to be investigated in eight cities, followed by a random selection of the households in these communities. Finally, we randomly selected interviewees from each household. The inclusion criteria were being over 18 years old and agreeing to participate in the survey voluntarily. A detailed introduction regarding the objectives as well as the privacy policy of this survey was provided to each participant recruited before informed consent was obtained. Ethical approval was obtained from all participating universities.

Although this data seems slightly outdated today, especially in the context of the pandemic, we continuously chose this data for two reasons. Firstly, this is a large-scale survey in China that has focused on the relationship between residents’ social capital and medical seeking behavior. As far as we know, there are no more current data on the same topic, even though the data is from 8 years ago. The questionnaires asked respondents about their medical experience and satisfaction with the medical process in the past year. This enabled us to conduct a more comprehensive measurement of the relationship between individual social capital and RSMS. Secondly, the data came from a wide range of cities, and the regional cultural characteristics and economic development levels that may affect the distribution of medical resources, were fully considered. The eight cities in the survey are located in East China, South China, Northwest China, Southwest China, Southeast China, Northeast China and North China. These include both cities with a higher level of economic development such as Shanghai, and with a lower level of economic development such as Lanzhou. The number of samples collected in each city is determined in a fixed proportion according to the total population of the city, and each city’s minimum sample collection is not less than 600. All the interviewees were selected by our strict random sampling, and we strictly checked and controlled the quality of the answers during the survey. Considering that this data has a wide range of urban sources and large sample size, and based on a strict random sampling method, the representativeness of this data for urban residents can be guaranteed to some extent. The 5,480 valid samples collected were further screened. Only samples with medical experience in the past year were retained to ensure that the evaluation of medical services was accurate and timely. Therefore, 3,207 samples were included in the final analysis.

### Main variables

The dependent variables were residents’ overall satisfaction with medical service, satisfaction with medical resources, and satisfaction with the medical system. As satisfaction cannot be directly observed, an attempt was made to translate subjective results into meaningful, quantifiable, and actionable data (Torrado and Blanca, 2022). We directly asked patients about their overall satisfaction with their healthcare experiences over the past year in a questionnaire. The questionnaire was originally a five-point scale, which was simplified as a dichotomous variable of satisfied and dissatisfied to evaluate overall patient satisfaction. Furthermore, referring to some mature scales (NEJM Catalyst, 2018), we inquired regarding satisfaction with medical safety, policies, costs, and resources based on their medical experiences in the past year. Using exploratory factor analysis, we aggregated these four indicators into two patient satisfaction factors with good consistency. Medical cost and resources referred to residents’ recognition and evaluation of medical resources, including medical costs, which was classified as residents’ satisfaction with medical resources (Amporfro et al., 2021). Medical safety and policies referred to trust and recognition of the overall medical system in the process of medical treatment, which was classified as residents’ satisfaction with the medical system (Natesan et al., 2019). For convenience of interpretation, the two patient satisfaction factors were scaled up equally from 0 to 100, and the analysis results are shown in Table 2.

**Table 2:** Factor analysis of satisfaction with medical resources and system (*N* = 3,207).

Factor type	Factor loadings	Factor indicators (cumulative proportion = 0.734)	
Satisfaction with medical resources		Proportion	0.394
Medical costs	0.890	Min/Max	0/100
Medical resources	0.869	Mean (Std)	32.386 (20.784)
**Satisfaction with the medical system**		**Proportion**	**0.341**
Medical safety	0.808	Min/Max	0/100
Medical policy	0.821	Mean (Std)	52.772 (15.099)

The independent variable was the use of individual social capital in the process of medical treatment. Individual social capital is usually measured in two different ways: based on the stock of individual social capital, which measures the presence of contacts and resources that may be helpful in the social network of residents, and based on the mobilization of individual social capital, which measures whether residents have actively used the connections and resources in the social network to achieve certain goals (Lin, 1999; Bian, 2018). In this study, individual social capital was measured from the perspective of its mobilization, and its stock was used as the control variable.

Considering the possible factors affecting individual social capital and RSMS (Gavurova et al., 2021; Lucadamo et al., 2021), this study used three types of control variables: social individual characteristics, including age, age squared, gender, marital status, education years, and log of family income, medical service, including insurance, service type, and trust, and the stock of individual social capital and social media variables, including social network resources, Guanxi cognition, and Internet use. Variable descriptive statistics are shown in Table 3.

**Table 3:** Variable descriptive statistics (*N* = 3,207).

Variable	Mean	Std	Min	Max	Instructions
Use individual social capital	0.285	0.451	0	1	1 = using individual social capital to seek medical services, 0 = not using it
Age	43.369	13.645	18	71	
Age square	2066.995	1228.076	324	5,041	
Gender	0.459	0.498	0	1	1 = male, 0 = female
Marital status	0.742	0.437	0	1	1 = married, 0 = unmarried or single
Education years	13.297	3.393	0	19	
Log of family income	2.112	0.684	0	5.303	
Medical insurance	0.766	0.423	0	1	1 = guaranteed, 0 = not guaranteed
Medical services types	0.515	0.500	0	1	1 = experience of hospitalization or surgery for serious illness, 0 = no such experience
Medical trust	0.607	0.488	0	1	1 = trust in the hospital, 0 = distrust in the hospital
Social networks resources	0.413	0.492	0	1	1 = there are medical staff in the new year network, 0 = no medical staff in it
Guanxi cognition	0.488	0.500	0	1	1 = guanxi is important in Chinese society, 0 = guanxi is not important
Internet use	0.646	0.478	0	1	1 = using internet social software, 0 = not using it

### Strategies for analysis

Dependent variables were dummy variables and factor scores, which are applicable to logistic and multiple linear regression models, respectively. Therefore, in the first step of the analysis, we established logistic and multiple linear regression models to test the individual social capital negative effect hypotheses, useless mechanism hypotheses, and erosion mechanism hypotheses.

However, the traditional regression method could not cope with the self-selection bias that may have existed in the relationship between individual social capital and RSMS. The propensity score method was utilized to alleviate the interference of this bias on the results. A series of confounding variables, such as economic status, education level, doctor-nurse relationship resources, and medical services, affect whether an individual chooses to use individual social capital. Moreover, these variables directly affect the generation of RSMS. Based on previous studies, propensity score weighting, referred to as inverse probability weighting (IPW) in Stata 17.0, and doubly robust estimator, referred to as augmented-IPW (AIPW) in Stata 17.0, can build a pair of experimental and control groups with differences only in the treatment variable (Austin and Stuart, 2015), like the individual social capital we used here. The interference of other variables on the treatment variables can then be reduced. Therefore, in the second step of the analysis, we controlled for self-selection bias by IPW and AIPW methods. We then conducted more robust verifications of the negative effect hypotheses, the useless mechanism hypotheses, and the erosion mechanism hypotheses.

Individuals may respond differently to the same behavior, and the effect of that behavior on individuals may systematically change due to their propensity to engage in that behavior. Therefore, in the final step of the analysis, a heterogeneous treatment effect model (Xie et al., 2012) was used to test the hypothesis of the differentiation mechanism. That is, for people with different possibilities (propensity values) of using individual social capital, will using individual social capital have different effects on their overall satisfaction with medical service?

## Results

Logistic and multiple linear regression models of individual social capital and RSMS were established, and the results are presented in Table 4.

**Table 4:** Logistic and multiple linear regression of Individual social capital and RSMS (*N* = 3,207).

	(M1)	(M2)	(M3)
Variable	Overall satisfaction	Satisfaction with medical resources	Satisfaction with medical system
Control variables	Y	Y	Y
Use Individual social capital	−0.215**	2.016**	−2.551***
	(0.092)	(0.869)	(0.613)
Constant	1.230**	22.827***	62.174***
	(0.515)	(4.889)	(3.400)
Pseudo R2 /R-squared	0.098	0.059	0.156

1. Robust standard errors in parentheses 2. ***p < 0.01, **p < 0.05 3. Control variables included age, age square, gender, marital status, education years, log of family income, medical insurance, medical services types, medical trust, social networks resources, Guanxi cognition, and Internet use.

The results of M1 showed that under the premise that other variables were unchanged, the overall satisfaction of residents who used individual social capital was significantly lower than that of residents who had not used it (*p* < 0.05). When individuals use individual social capital to seek medical services, the occurrence rate of overall satisfaction with medical service was only equivalent to 80.697% of those who did not use individual social capital. This indicates that residents with individual social capital were more likely to give negative evaluations of overall satisfaction. Hypothesis 1.1 was supported by the data, which were consistent with previous empirical results (Ruan, 2017).

The results of M2 and M3 demonstrated satisfaction with medical resources among residents who sought medical services through individual social capital, which was 2.016 higher (*p* < 0.01) than for those who did not, and satisfaction with the medical system was 2.551 lower (*p* < 0.05). Individual social capital had a positive effect on satisfaction with medical resources and a negative effect on satisfaction with the medical system. Thus, Hypotheses 3.1 and 3.2 were tentatively supported.

IPW and AIPW methods were used to strictly control the self-selection bias between individual social capital and RSMS, and the results are shown in Table 5.

**Table 5:** IPW and AIPW of Individual social capital and RSMS (*N* = 3,207).

Variable	(M4) AIPW	(M5) IPW	(M6) AIPW	(M7) IPW	(M8) AIPW
	Overall satisfaction	Satisfaction with medical resources	Satisfaction with medical resources	Satisfaction with medical system	Satisfaction with medical system
Control variables	Y	Y	Y	Y	Y
Control group mean	0.527***	31.810***	31.822***	53.464***	53.479***
ATE	−0.068***	2.227**	2.290**	−3.072***	−2.775***
(Used vs. not used)	(0.023)	(1.005)	(0.995)	(0.727)	(0.717)
ATT		2.025**		−2.433***	
(Used vs. not used)		(0.911)		(0.683)	

1. Overall patient satisfaction outputs only double robust estimates with logistic as the link function of the outcome model. 2. Robust standard errors in parentheses. 3. ***p < 0.01, **p < 0.05 4. The control variables are the same as in Table 4. All control variables are included in the selection model, and the balance test shows that the significant differences in each confounding variable between the experimental group and the control group have been eliminated.

M4 indicates that after the control for self-selection bias, the absolute value of the influence coefficient of individual social capital use on overall satisfaction decreased; however, it was significantly negative (*p* < 0.01). Individual social capital exhibited significant negative effects that impaired overall satisfaction. The negative effort hypothesis, including Hypotheses 1.1 and 1.2, was supported, and the negative function of individual social capital on RSMS was further confirmed after overcoming the self-selection bias.

M5 and M6 indicated that the ATE effect of using individual social capital on patient medical resource satisfaction was significantly positive (*p* < 0.01). The positive effect of individual social capital on satisfaction with medical resources was verified. Hypothesis 3.1 was supported, whereas Hypothesis 2.1 was not. M7 and M8 showed that the ATE effect of using individual social capital on medical system satisfaction was significantly negative (*p* < 0.01). The negative effect of individual social capital on satisfaction with the medical system was verified. This supported Hypotheses 2.2 and 3.2.

The results of M5-M8 jointly supported the erosion mechanism hypothesis, whereas the useless mechanism hypothesis was not supported. The use of individual social capital helped residents obtain better medical resources and lower medical costs, which improved their satisfaction with medical resources. However, this undermined confidence with the medical system and safety, resulting in decreased satisfaction with the medical system. The negative influence of individual social capital on medical satisfaction was more likely to originate from the instrumental behavior of residents to actively obtain resources rather than the irrational behavior of blind conformity, despite potential harm to the medical system. The erosion mechanism, rather than the useless mechanism, was closer to the negative function of individual social capital in the process of medical treatment.

**Figure 1:**
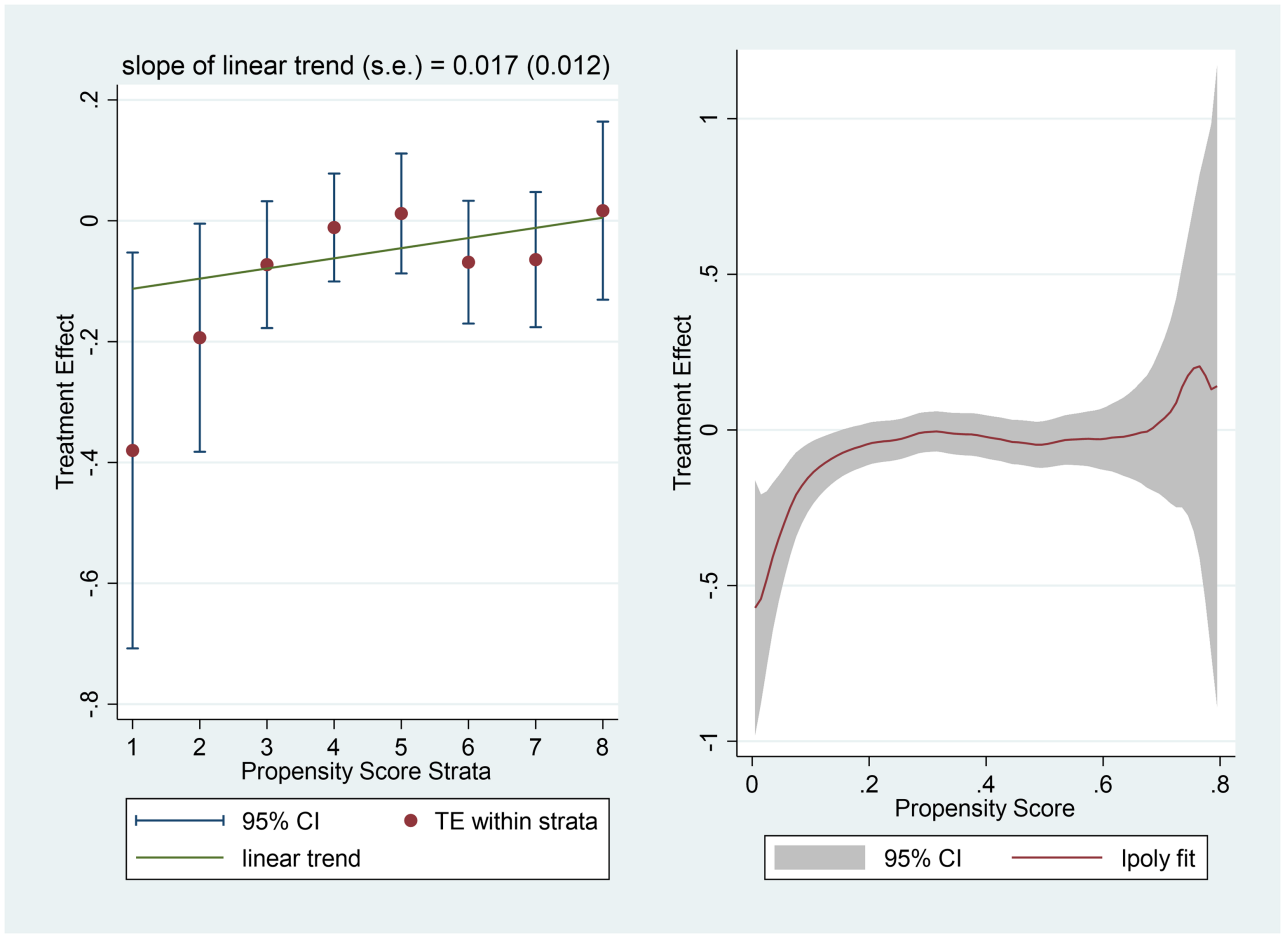
Heterogeneous treatment effects of Individual social capital and overall satisfaction with medcial services (N=3207).

Finally, a heterogeneous treatment effect model was used to verify the differentiation mechanism hypothesis (Figure 1).

The control variables were the same as those presented in Table 4. To ensure the robustness of the results, we used two evaluation strategies: Hierarchical-multilevel (HM) and Smoothing-differencing (SD) methods. These methods yielded consistent findings. The influence coefficient of individual social capital on overall satisfaction tended to be positive with the expansion of the propensity value. With the increase in the possibility of using Individual social capital, the negative effect on overall satisfaction decreased. For patients with a higher likelihood of using individual social capital, the negative impact on overall satisfaction was significantly smaller. However, for patients with a low possibility of using individual social capital, the negative impact on overall satisfaction was more pronounced. This indicated that the negative function of individual social capital was different for people with different possibilities for individual social capital usage in the process of medical treatment. The differentiation mechanism hypothesis was supported.

## Discussion

This study confirmed the influence of individual social capital on RSMS and investigated the mechanism of this negative effect, while trying to overcome the self-selection bias. Although some studies have noted that the use of individual social capital may harm the process of medical service (Zou et al., 2018; Zhang et al., 2021), due to the lack of large-scale questionnaire data support, this conclusion lacks sufficient representativeness and universality. More importantly, the mechanisms of this negative effect must be understood to promote research on individual social capital and RSMS. Using large-scale questionnaire data and a series of quantitative methods, three findings were revealed, which supported the negative effect hypotheses, the erosion mechanism hypotheses, and the differentiation mechanism hypotheses, whereas the useless mechanism hypothesis was not supported.

First, after excluding the interference of confounding variables, the results indicated that the use of individual social capital in medical treatment caused a robust and negative impact on satisfaction. Residents who sought medical services through individual social capital were more likely to give negative evaluations of their medical experience, leading to lower overall satisfaction with medical service, than those who did not. Similar to previous studies based on qualitative methods (Zhang et al., 2021), this study reconfirmed the important and usually negative influence of individual social capital on residents’ satisfaction and medical services considering self-selection bias. When seeking medical services through individual social capital becomes a cultural habit under the constraints of Guanxi determinism, such a capital becomes an important psychosocial factor affecting residents’ satisfaction with medical service systems. Moreover, in societies where formal medical rules are inadequate, or medical resources are scarce, as is the case in most East-Asian developing countries similar to China, informal individual social capital operations may tend to be active, and become a key psychosocial factor influencing residents’ satisfaction with health care services. The influence of this social context-related psychosocial factor should not be overlooked in the evaluation of health delivery systems.

In addition, the erosion mechanism, rather than the useless mechanism, was more likely to cause the negative effect of Individual social capital on patient satisfaction. The use of Individual social capital eroded residents’ confidence in medical policies and safety, leading to a significant decline in satisfaction with the medical system. However, the use of Individual social capital may reduce medical costs, help residents obtain better medical resources, and significantly improve satisfaction with medical resources. Therefore, Individual social capital provided resource advantages while creating doubts regarding the medical system and concerns about future medical situations. This result suggests that, seeking medical care through individual social capital is not always futile. Contradictorily, this behavior can frequently lead to definite benefits, such as more appropriate treatment options and better quality of care. Despite the widespread cultural pressures in social space, individual social capital’s usage is not exclusively a patient’s cultural burden for patients, but may also be a rational choice with certain benefits. In addition, the use of individual social capital is associated with the erosion of confidence in the health care system. This connection is important in the construction of the doctor-patient relationship, and the improvement of the health care system.

Moreover, medical experiences and satisfaction of individuals with different social capital use possibilities were variably affected by the use of Individual social capital. For individuals with a lower probability of using Individual social capital, deciding to use Individual social capital was often passive or even forced. They faced greater difficulties and incurred higher costs, leading to a further reduction in satisfaction. As many residents lack a strong individual social capital mobilization ability, the differentiation mechanism and resulting inequality of Individual social capital may cause widespread negative influence of Individual social capital in the field of medical treatment (Rasmussen et al., 2021). If we consider RSMS as a valid indicator to evaluate the healthcare system, equal to other indicators, such as residents’ choice of medical institutions (He et al., 2022). The significant influence of individual social capital on the medical process may lead to negative consequences, causing a general crisis of confidence and moral pressure on China’s medical service system. And more importantly, seeking healthcare through individual social capital is a common and dynamic phenomenon in Chinese society which is often accompanied by great disputes and challenges. As one of the important sources of life satisfaction, RSMS may intensify residents’ perception of social injustice and relative deprivation (Jia et al., 2020), and bring long-term pressure and challenges to residents’ life satisfaction and subjective well-being if it is continuously negatively affected by individual social capital inequality.

Different from previous studies, we further considered the mechanism and heterogeneous distribution of individual social capital’s negative impact on RSMS. Through a more detailed examination of residents’ different evaluations of the medical system and medical services, this study emphasized the individual rational basis behind the active use of individual social capital. Although individual social capital is closely related to China’s Guanxi culture and social context, its operation hides a rational basis and instrumental orientation. In previous studies, the abuse of individual social capital in the health service system was often attributed to the irrational choices and moral corruption of doctors and patients (Cheng and Zou, 2015). Individual social capital was considered as having no practical role and as a cultural burden that added expenses. This view is consistent with the moral criticism and negative image that individual social capital operation often encountered in Chinese society (Gold, 1985). By contrast, the results of this study showed the motivation behind the operation of individual social capital in medical service is similar to all other aspects (Cao et al., 2022). Residents are not powerless mobs when faced with the choice of using individual social capital. Rather, the advantages of medical resources that individual social capital provides for residents outside the formal institutional constraints attract them to mobilize it. Although the damage to the medical system is self-evident, the rational and instrumental motivation hidden in the behavioral choice of users of individual social capital should not be ignored.

Furthermore, this study examined the unity of the positive and negative functions of individual social capital. The positive and negative aspects of individual social capital were simultaneously observed, with differences. Specific social capital that facilitates one action may be useless or even harmful to others (Coleman, 1990). There is no clear line between the positive and negative sides of individual social capital. However, the manner in which individuals use the resource advantages provided by individual social capital, and its effects, are significant. Individual social capital is a double-edged sword for users, and the positive side of resource advantages is often accompanied by the negative aspect of institutional damage. Moreover, when different people navigate this double-edged sword, the negative and positive aspects differ. For individuals with a low probability of using individual social capital due to factors such as personality and ability, its use means greater pressure and challenges for these individuals, and it is difficult to generate corresponding rewards. The spread of individual social capital in seeking healthcare may lead to serious medical and long-term health inequality, especially for people who do not have sufficient income, good education, and strong social networks.

The findings of this study provide warnings for the development of future medical service systems. Social capital has a space to operate in the medical services area because of fierce resource competition and a high professional threshold. Access to medical services through social capital is not only applicable to China but may be widespread in other countries, especially developing countries in East Asia (Li and Guo, 2022). The negative function of individual social capital shows that, it is undoubtedly crucial to limit the abuse of such capital in the medical field. But the findings of this study suggest that, achieving this goal may encounter more challenges than we previously thought. On the one hand, the pursuit of medical services through individual social capital has a sufficient basis for rational action, which is difficult to solve using a simple moral constraint mechanism. Contradictorily, there is a clear difference in the experience of using individual social capital in the process of medical treatment between individuals. This indicates the higher difficulty in developing a consensus to resist the use of individual social capital. Based on our findings, possible solutions to this problem are to eliminate the rational basis for the operation of individual social capital, as much as possible. It requires the joint efforts of doctors, patients, and the government. Doctors need to establish an equal doctor-patient relationship, instead of giving preferential treatment to those who use individual social capital. Patients should have reasonable trust on doctors’ professional quality and avoid imposing unreasonable expectations and pressures on doctors, and make a fair evaluation of medical services on the premise of not considering individual social capital. The government must be particularly vigilant against the destruction of medical rules and institutions, through the proliferation of individual social capital. Moreover, it is necessary to continuously optimize the fair distribution of medical resources and establish a complete and sound medical system. The medical system needs to provide all residents, including the disadvantages groups, with the same medical services, no matter what kind of individual social capital they have.

This study had some limitations that deserve further improvements. First, the research data used in this study were nearly 8 years old. With the continuous improvement of China’s social medical service system, the relationship between individual social capital and RSMS may have undergone some recent changes. Although the role of individual social capital in Chinese society has been relatively stable, the research conclusions may be representative today. However, more recent data should be used in future studies, especially data after the COVID-19 outbreak, as it may change the distribution pattern of medical resources and the role of individual social capital. At present, we are conducting an updated questionnaire survey, to collect the above data, but due to the disturbance of the epidemic, this survey project that started in 2019, has not yet been completed. After the project’s completion, we may be able to give a better answer to the above questions. In addition, this study did not consider the interaction between medical service type and individual social capital. The use of individual social capital is often more urgent and necessary for patients with serious diseases, such as cardiovascular disease, than those with mild diseases, such as a cough. The impact of these differences on RSMS should be further investigated in future studies. Especially for research in some medical fields, under the huge social impact brought by the epidemic, it may become increasingly important to jointly consider patients’ psychosocial factors and physiological factors, in evaluating medical quality.

## Data availability statement

The datasets presented in this article are not readily available because the sharing of data requires the consent of all participating universities. Requests to access the datasets should be directed to wangwb@jlu.edu.cn.

## Ethics statement

According to the local legislation and institutional requirements, ethical review and written informed consent were not required.

## Author contributions

WW and YC: conceptualization. YC: methodology and writing—original draft preparation. WW: validation, writing—review and editing, supervision, and project administration. All authors contributed to the article and approved the submitted version.

## Conflict of interest

The authors declare that the research was conducted in the absence of any commercial or financial relationships that could be construed as a potential conflict of interest.

## Publisher’s note

All claims expressed in this article are solely those of the authors and do not necessarily represent those of their affiliated organizations, or those of the publisher, the editors and the reviewers. Any product that may be evaluated in this article, or claim that may be made by its manufacturer, is not guaranteed or endorsed by the publisher.
